# Correlation between Treatment Outcomes and Serum Vitamin D Levels As Well As Infliximab Trough Concentration among Chinese Patients with Crohn's Disease

**DOI:** 10.1155/2023/6675401

**Published:** 2023-10-06

**Authors:** Xiaomei Song, Huihui Zhang, Hao Wang, Zhongyue Li, Xiaoqin Zhou, Hong Guo

**Affiliations:** ^1^Department of Gastroenterology, Chongqing General Hospital, Chongqing, China; ^2^Department of Gastroenterology, Children's Hospital of Chongqing Medical University, National Clinical Research Center for Child Health and Disorders, Ministry of Education Key Laboratory of Child Development and Disorders, Chongqing Key Laboratory of Pediatrics, Chongqing Key Laboratory of Child Health and Nutrition, Chongqing, China

## Abstract

**Background:**

The relationship between vitamin D (vit-D) levels and the effectiveness of infliximab (IFX) in patients with Crohn's disease (CD) remains controversial.

**Objective:**

To evaluate the interaction between vit-D levels and the response to IFX therapy in patients with CD.

**Methods:**

This was a retrospective cohort study. Serum vit-D and IFX trough concentrations (TC) were measured in 84 patients, and statistical analyses were performed.

**Results:**

The total vit-D deficiency rate at enrollment, at week 14 and week 38, was 64.3%, 41.67%, and 37.5%, respectively (*P* < 0.001). CD activity index (CDAI) (120, range, 93–142.75) and simplified endoscopic activity score for CD (SES-CD) (2, range, 0–4) at week 14 were lower than that of enrollment (CDAI, 136.5, range, 101.25–196; SES-CD 13, range, 5–23) (*P* < 0.001). The biochemical remission (BR), clinical remission (CR), endoscopic remission (ER), and response (ERe) rates of week 38 were 76.1%, 88.5%, 22.4%, and 67.2%, respectively. vit-D levels at enrollment were positively correlated with CDAI at week 38 (*P* = 0.024). IFX serum TC was related to BR (*P* = 0.036), CR (*P* = 0.032) at week 14, and ERe (*P* = 0.009) at week 38.

**Conclusion:**

Among Chinese patients with CD, vit-D levels prior to IFX therapy are related to CDAI scores, and IFX serum TC is associated with BR, CR, and ERe.

## 1. Introduction

Inflammatory bowel disease (IBD) is a chronic inflammatory disease of the intestine that encompasses ulcerative colitis (UC) and Crohn's disease (CD). Clinical symptoms include chronic or subacute abdominal pain, diarrhea, bloody stool, fever, oral ulcers, crissum abscess or fistula, arthritis, and skin rashes. Recurrent disease can cause anemia, weight loss, and malnutrition, which seriously affect patients' quality of life and impose an economic burden and psychological pressure [1, 2]. According to the reported global incidence of IBD [3], the highest incidence of UC is 5.05/1000, and that of CD is 3.22/1000. In China, the total number of IBD cases from 2005 to 2014 was approximately 350,000, and it is expected to reach 1.5 million by 2025 [4]. In genetically susceptible individuals, a dysregulated immune response to intestinal flora and environmental factors has been proposed as the pathogenic mechanism in the incidence and progression of IBD [5].

Anti-tumour necrosis factor-*α* (anti-TNF*α*) biological therapies have dramatically reduced surgery and hospitalization rates while simultaneously improving the quality of life in patients with IBD [6]. Although TNF*α* inhibitors are highly effective, nearly 30% of patients lose their therapeutic response, partly due to antidrug antibody production and inadequate drug concentrations [7, 8]. Vitamin D (vit-D) is a nutrient that plays an important role in the pathogenesis of IBD [9, 10]. Recent research has shown that serum vit-D levels impact anti-TNF*α* therapy. A study in pediatric patients with IBD found that vit-D insufficiency before anti-TNF*α* treatment resulted in a poor response to induction therapy [11]. Zator et al. demonstrated an association with earlier cessation of anti-TNF*α* therapy in patients with IBD who had insufficient vit-D levels before initiation of therapy, suggesting that vit-D may be an important auxiliary treatment to anti-TNF*α* therapy [12]. However, this remains controversial. Reich et al. demonstrated that patients with low serum vit-D before initiating therapy reached a higher proportion of clinical remission (CR) after induction doses of infliximab (IFX) [13].

The current study is aimed at establishing the association of clinical outcomes with serum vit-D and a possible correlation with IFX serum trough concentration (TC) and anti-TNF*α* antibody (ATI) levels, or the lack thereof.

## 2. Materials and Methods

This retrospective cohort study was conducted at Chongqing General Hospital. The study involved 38 weeks of follow-up and was conducted from January 2019 to May 2021. Written informed consent was obtained from all patients, and the study was approved by the ethics committee of the Chongqing General Hospital (ethics review number: KY-S2022-023-01).

### 2.1. Study Design

**Figure 1:**
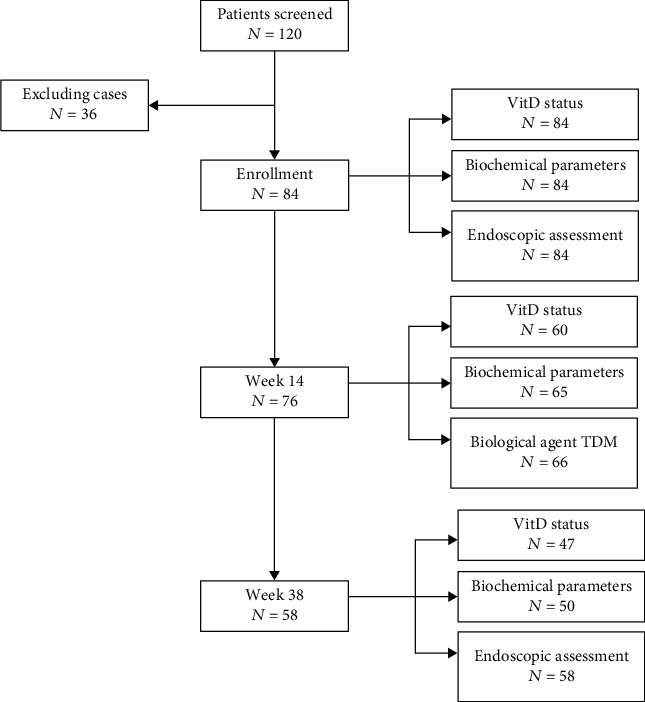
Flow diagram. Vit-D: vitamin D; week 14: 4th IFX treatment; week 38: 7th IFX treatment; TDM: therapeutic drug monitoring.

Patients with available data at enrollment were included and followed up at the week 14 IFX treatment and the week 38 IFX treatment. Demographic and clinical characteristics were collected at enrollment, and nutritional indices (including vit-D levels) and biochemical parameters were collected at all three-time points. Additionally, data on clinical outcomes, including biochemical remission (BR) and CR, were collected at week 14 and week 38, and endoscopic response and endoscopic remission were collected at week 38. In addition, serum IFX concentrations were collected at week 14. Correlation analysis was carried out among vit-D levels, serum IFX concentrations at week 14, and clinical outcomes (Figure 1).

### 2.2. Patient Population

Inclusion criteria were confirmed with CD according to the Chinese consensus on the diagnosis and treatment of IBD (2018, Beijing) [14] and according to the guidelines for the management of IBD in adults [15]. The diagnosis of CD was based on standard criteria, including the combination of clinical symptoms, endoscopy, radiology, pathology, and surgical history. The main treatment plan was IFX therapy, without vit-D supplementation, with complete blood biochemical parameters and nutritional indices, as well as week 14 biological agent serum concentration monitoring (IFX-TC and IFX-ATI) and week 38 endoscopic assessment. Patients with vit-D supplementation and incomplete multiple blood biochemical parameters or incomplete endoscopic evaluation were excluded. The final CD cohort consisted of 84 patients.

### 2.3. Vit-D Assessments

Vit-D data prior to IFX therapy was available for 84 patients. The cut-off concentration for vit-D deficiency was based on the Endocrine Society Clinical Practice Guideline 2011 [16]; patients with vit-D levels < 20 ng/mL were considered vit-D deficient, those with >30 ng/mL were classified as vit-D sufficient, and those with 20-30 ng/mL were classified as vit-D insufficient. Peripheral blood vit-D was detected using chemiluminescence in the laboratory of Chongqing General Hospital.

### 2.4. IFX Concentration

Therapeutic concentration monitoring of IFX was performed at week 14 using a fluorescence immunochromatography IFX detection kit (Suzhou Herui BioMed Co., Ltd.) at the Suzhou Herui IBD Diagnostic Technology Research Center. Concentrations > 3 *μ*g/mL were considered sufficient [14]. ATI < 20 ng/mL was defined as negative [17]. The ATI detection kit (Suzhou Herui BioMed Co., Ltd.) is a quantitative fluorescence immunochromatographic assay tool.

### 2.5. Classification and Definition

The extent of the disease was defined using the Montreal classification [18]. BR was defined as C-reactive protein (CRP) < 5 mg/L and erythrocyte sedimentation rate (ESR) < 20 mm/h [19]. CR was defined as a CD activity index (CDAI) < 150 [20]. Endoscopic assessment was performed at the clinical endpoint (38 weeks), with endoscopic remission defined as a simplified endoscopic activity score for CD (SES-CD) < 4 and endoscopic response as a 50% reduction in the SES-CD score from baseline [21].

### 2.6. Statistical Analyses

All statistical tests were performed using SPSS software version 25.0 (SPSS, Chicago, IL, USA) and GraphPad Prism version 5 (GraphPad Software, San Diego, CA). For quantitative variables, data are shown as the mean ± standard deviation, or as the median and interquartile range (IQR), according to the presence or absence of a normal distribution, respectively. Categorical variables are expressed as percentages. Student's *t*-test and the Mann–Whitney *U* test were used to compare independent continuous variables, whereas the paired *t*-test and the Wilcoxon signed-rank test were used to compare dependent continuous variables. Categorical variables were compared using the chi-square test and rank-sum test. Linear regression was used to determine the independent factors associated with the CDAI outcomes. All *P* values < 0.05 in the final multivariate model were considered statistically significant.

## 3. Results

### 3.1. Demographic and Clinical Characteristics

There were 84 patients enrolled. The mean ages of the patients were 26 (22–34). The male/female ratio was 2.5 : 1. The proportion of patients with low body weight was 52.4%. The local patients accounted for 86.9%. 54.8% were employed, mainly indoors. 76.2% of the patients did not smoke, and 59.5% had college degrees or higher qualifications. Abdominal pain and diarrhea were the main clinical manifestations. The positive intestinal surgery history and perianal surgery rates were 75% and 56%, respectively. Immunosuppressant (IMM) exposure history was 36.9%, and azathioprine was the most important IMM type, accounting for 90.0%. According to the Montreal classification standard [18], A1 accounted for 4.7%, A2 for 81.0%, and A3 for 14.3%. In terms of disease range, L1 accounted for 23.8%, L2 for 11.9%, L3 for 56.0%, L3 + L4 for 7.1%, and L2 + L4 for 1.2%. For disease behavior, B1 accounted for 53.6%, B2 for 31.0%, and B3 for 15.5%. Perianal lesions were positive in 65.5% (Table 1).

### 3.2. Biochemical Parameters, Nutritional Indices, and Clinical Assessment

Table 2 presents the main clinical indices of enrollment, week 14, and week 38. Analysis of the peripheral blood revealed that vit-D levels were significantly higher at week 14 (21.8, range, 17.0–25.7) and week 38 (21.1, range, 16.2–27.7) than at enrollment (16.2, range, 9.6–21.9) (*P* < 0.001). Vit-D deficiency rates at enrollment, week 14, and week 38 were 64.3%, 41.7%, and 37.5%, respectively (*P* = 0.009). Inflammatory indices, including CRP, high-sensitive CRP (hs-CRP), ESR, and platelet (PLT) of week 14 and week 38, were lower than those of enrollment (*P* < 0.001). The nutritional indexes, hemoglobin (Hb), prealbumin (PA), and albumin (ALB), improved with week 14 and week 38 (*P* < 0.001). Clinical evaluation includes CDAI, SES-CD, BR, CR, endoscopic remission, and response rate. CDAI of week 38 (120, range 93–142.8) was lower than that of enrollment (136.5, range 101.3–196) (week 38 vs. enrollment, *P* = 0.01). SES-CD of week 38 (2, range 0–4) was lower than that of enrollment (13, range 5–23) (*P* < 0.001). BR, CR, endoscopic remission, and response rate of week 38 were 76.1%, 88.5%, 22.4%, and 67.2%, respectively (Table 2).

#### 3.2.1. Vit-D Concentrations and Clinical Outcomes

The correlation analysis of the clinical index (including biochemical parameters and nutritional indices) of enrollment and clinical evaluation (including CDAI, SES-CD, BR, CR, endoscopic remission, and response rate) of week 14 and week 38 revealed that only vitamin D had a definite correlation. Vit-D level at enrollment (*r* = −.369, *P* = 0.007), week 14 (*r* = −.326, *P* = 0.035), and week 38 (*r* = −.307, *P* = 0.043) were correlated with the CDAI at week 38. Linear regression analysis revealed that the enrolled vit-D level was negatively correlated with CDAI at week 38 (*b* = −1.378, *P* = 0.024) (Table 3).

The 84 patients included in enrollment were divided into deficiency (*n* = 54), insufficiency (*n* = 23), and sufficiency groups (*n* = 7), according to the level of vit-D. BR (effective data, 60 patients) and CR (77 patients) were evaluated at week 14. The BR rates were 79.60%, 77.8%, and 100% (*P* = 1), and the CR rates were 70.4%, 68.8%, and 66.7% (*P* = 1) in the three groups, respectively. Similarly, BR (47 patients), CR (52 patients), endoscopic remission (58 patients), and endoscopic response (58 patients) were evaluated at week 38, and there was no statistical difference in the clinical outcomes of the deficiency, insufficient, and sufficient groups (Table 4).

#### 3.2.2. IFX Concentrations and Clinical Outcomes

For week 14, the following were observed: 10 patients (15.1%) with sufficient IFX concentrations, 24 (36.4%) with efficient IFX concentrations, and 32 (48.5%) with insufficient IFX concentrations. IFX-TC was associated with the biochemical (*P* = 0.036) and CRs (*P* = 0.032) at week 14, and endoscopic response at week 38 (*P* = 0.009). The difference was statistically significant (Table 5). The negative ATI rate for week 14 was 62 (93.9%). However, ATI at week 14 was not associated with the clinical outcomes of week 14 and week 38 (*P* > 0.05) (supplementary data).

## 4. Discussion

The correlation between vit-D levels and IFX-TC has not yet been extensively explored. This study analyzed the relationship between vit-D nutritional status, serum IFX concentration, and clinical outcomes to determine the long-term treatment outcomes in patients with CD. We found that vit-D deficiency is common in patients with CD. Low vit-D levels prior to IFX treatment are negatively correlated with CDAI scores, and IFX serum concentrations are significantly associated with clinical outcomes.

Vit-D deficiency in patients with IBD is currently considered detrimental, and several studies have hypothesized that vit-D is related to IBD outcomes. A meta-analysis has previously demonstrated that vit-D levels are inversely related to CD and UC [22]. Furthermore, vit-D normalization is associated with a reduced risk of relapse [23–25]. In our study, the total vit-D deficiency rate was 64.3%. Additionally, the advancement of CD treatment with IFX gradually improves vit-D levels. We also observed that lower baseline vit-D levels led to higher CDAI, despite IFX treatment. However, vit-D levels did not impact clinical outcomes, including BR, CR, endoscopic remission, and endoscopic response rates. The lack of effect on clinical outcomes may be attributable to the small sample size or the insufficient degree of change in CDAI to influence disease activity and the remission period. At present, we are expanding the sample size and conducting multicenter studies to clarify the relationship between vit-D and clinical outcomes.

As the most classic biological agent for CD treatment, IFX has maximal therapeutic effects and maintains high CR and endoscopic remission rates, which are essential for clinical treatment. Monitoring anti-TNF*α* levels helps control serum drug levels, predict long-term clinical outcomes, optimize treatment, and decrease the economic burden on patients. Several studies have independently demonstrated a correlation between IFX-TC and IBD disease status [26, 27]. In this study, we monitored the concentration of IFX and evaluated its relationship with CR, endoscopic remission, and endoscopic response. Patients with sufficient serum concentrations had a higher BR, CR, and endoscopic response rate. Our findings are consistent with those of a systematic review and meta-analysis that revealed a significant difference between serum IFX levels in patients with IBD in remission and those patients who relapsed with a trough threshold of >2 *μ*g/mL during maintenance associated with a greater probability of CR and mucosal healing. In addition, IFX-ATI did not influence CR, endoscopic remission, or endoscopic response.

There are some limitations to the study. First, the sample size was small. Second, we could not measure the disease activity of all patients by endoscopy or radiological examination. Third, the lack of dietary intake monitoring could affect the nutritional status assessments. Finally, there may be a need for a more extensive follow-up time to accurately determine clinical outcomes.

## 5. Conclusions

Vit-D levels prior to IFX therapy are related to CD activity index scores, and IFX-TCs are associated with the outcomes of patients with CD.

## Figures and Tables

**Table 1:** Demographic characteristics.

Variables	
Number of patients	84
Age (y)	26 (22–34)
Gender	
Female (%)	24 (28.60)
Male (%)	60 (71.43)
Height (m) (mean ± SD)	1.68 ± 0.08
Weight (kg) (mean ± SD)	52.86 ± 8.81
BMI	
Underweight (%)	44 (52.38)
Normal weight (%)	39 (46.43)
Preobese (%)	1 (1.19)
Obese (%)	0
Provinces	
Chongqing (%)	73 (86.90)
Sichuan (%)	9 (10.71)
Others (%)	2 (2.38)
Employment situation	
Unemployed (%)	38 (45.24)
Employed (%)	46 (54.76)
Occupation	
Indoor (%)	79 (94.05)
Outdoor (%)	5(5.95)
Marital status	
Unmarried (%)	49 (58.33)
Married (%)	33 (39.29)
Divorced (%)	3 (3.57)
Smoking status	
No (%)	64 (76.19)
Yes (%)	20 (23.81)
Education background	
Primary school, *n* (%)	1 (1.19)
Middle school (%)	9 (10.71)
Senior high school (%)75	24 (28.57)
University and above (%)	50 (59.52)
Course of disease mean (IQR)/*N*	2.99 (1.64–5.56)/84
Clinical symptoms	
Abdominal	51 (60.71)
Diarrhea	35 (41.67)
Hematochezia	6 (7.14)
Crissum diseases	4 (4.76)
Extraintestinal manifestations	6 (7.14)
Fever	2 (2.38)
Intestinal surgery, *n* (%)	
No	21 (25.00)
Yes	63 (75.00)
Perianal surgery, *n* (%)	
No	37 (44.05)
Yes	47 (55.95)
IMM	
No	53 (63.10)
Yes	31 (36.90)
IMM type	
Azathioprine	27 (90.00)
Methotrexate	2 (6.67)
Thalidomide	1 (3.33)
Diagnosis age, *n* (%)	
A1 ≤ 16 y	4 (4.76)
A2 17~40 y	68 (80.95)
A3 > 40 y	12 (14.29)
Disease extent, *n* (%)	
L1	20 (23.81)
L2	10 (11.90)
L3	47 (55.95)
L3 + L4	6 (7.14)
L2 + L4	1 (1.19)
Behavior, *n* (%)	
B1	45 (53.57)
B2	26 (30.95)
B3	13 (15.48)
Perianal lesions	
No	29 (34.52)
Yes	55 (65.48)

y, year; m, month; SD, standard deviation; kg, kilogram; BMI, body mass index; IQR, interquartile range; IMM, immunosuppressant.

**Table 2:** Biochemical parameters, nutritional indices, and clinical assessment at three follow-up time points.

Time point	Vit-D status		Biochemical parameters				CDAI	SES-CD	Biochemical remission	Clinical remission	Endoscopic remission	Endoscopic response
			Inflammatory indices IQR/N		Nutritional indices							
Enrollment	IQR/N 16.15 (9.55~21.875)/84		CRP	22.1 (5~61.3)/84	Hb	114.5 (92.3~127.5)/84	136.5 (101.25~196)/84	13 (5~23)/84	/	/	/	/
	Sufficiency, *n* (%)	7/84 (8.3)	hs-CRP	27.2 (6.5~62.3)/84	PA	157 (116~212.8)/84						
	Insufficiency, *n* (%)	23/84 (27.4)	ESR	22 (12.25~40)/84	ALB	38.9 (35.8~43.9)/84						
	Deficiency, *n* (%)	54/84 (64.3)	PLT	382.5 (300.8~461.8)/84	/							

Week 14	IQR/N 21.8 (16.95~25.65)/60^∗∗^		CRP	5 (3.1~5)/59	Hb	142 (133~151)/65^∗∗∗^	137.5 (114~166.5)/76	/	48/60 (80)	53/76 (69.7)	/	/
	Sufficiency, *n* (%)	7/60 (11.67)	hs-CRP	0.99 (0.4~5.3)/65^∗∗∗^	PA	259 (216~298)/63^∗∗∗^						
	Insufficiency, *n* (%)	28/60 (46.7)	ESR	5 (3~10.5)/65^∗∗∗^	ALB	48.3 (44.5~50.9)/65^∗∗∗^						
	Deficiency, *n* (%)	25/60 (41.67)	PLT	246 (213.5~287)/65^∗∗∗^	/							

Week 38	IQR/N 21.1 (16.2~27.7)/47^∗∗∗^		CRP	5 (3.7~6.6)/47^∗∗∗^	Hb	144 (126.5~152.25)/50^∗∗∗^	120 (93~142.75)/52^∗∗^	2 (0~4)/58^∗∗∗^	35/46 (76.1)	46/52 (88.5)	13/58 (22.4)	39/58 (67.2)
	Sufficiency, *n* (%)	9/47 (19.12)	hs-CRP	1.84 (0.4~8.1)/49^∗∗∗^	PA	250 (219.75~297.5)/50^∗∗∗^						
	Insufficiency, *n* (%)	20/47 (42.55)	ESR	5.6 (3~12)/49^∗∗∗^	ALB	46.6 (45.1~50.0)/50^∗∗∗^						
	Deficiency, *n* (%)	18/47 (38.3)	PLT	260 (209.3~308.3)/50^∗∗∗^	/							

Vit-D: vitamin D; CDAI: Crohn's disease activity index; SES-CD: simplified endoscopic activity score for Crohn's disease; IFX: infliximab; IQR: interquartile range; CRP: C-reactive protein; hs-CRP: high-sensitive C-reaction protein; ESR: erythrocyte sedimentation rate; PTL: platelet; Hb: hemoglobin; PA: prealbumin; ALB: albumin. Week 14 vs. enrollment vs. enrollment. ^∗∗^*P* < 0.01; ^∗∗∗^*P* < 0.001.

**Table 3:** The influence of vitamin D levels at three-time points on Crohn's disease activity index at the week 38 IFX treatment in Crohn's disease patients (linear regression, enter) (*n* = 84).

		Unstandardized coefficients	Std. error	Standardized coefficients	*t*	Sig.	VIF
(Constant)		295.756	54.415		5.435	0	
Vit-D status	Enrollment	-1.378	0.583	-0.324	-2.363	0.024	1.086
	Week 14	-0.929	0.599	-0.224	-1.549	0.13	1.206
	Week 38	-0.662	0.551	-0.181	-1.2	0.238	1.31
*F*				5.681			
*P*				0.001			
*R* ^2^				0.394			
Dependent variable: CDAI (IFX7)							

Vit-D: vitamin D; CDAI: Crohn's disease activity index; VIF: variance inflation factor.

**Table 4:** The influence of vitamin D level at enrollment on clinical outcomes at the week 14 and week 38 IFX treatment in Crohn's disease patients.

Time point	Outcomes	Vit-D level at enrollment			Chi-square	*P*
		Deficiency	Insufficiency	Sufficiency		
Week 14	Biochemical remission	39/49 (79.60)	7/9 (77.80)	2/2 (100)	0.409	1
	Clinical remission	38/54 (70.40)	11/16 (68.80)	4/6 (66.70)	0.239	1

Week 38	Biochemical remission	34/44 (77.30)	1/2 (50)	0/1 (0)	3.725	0.156
	Clinical remission	34/40 (85)	7/7 (100)	5/5 (100)	0.932	0.774
	Endoscopic remission	29/39 (74.40)	11/14 (78.60)	5/5 (100)	1.197	0.689
	Endoscopic response	27/39 (69.20)	8/14 (57.10)	4/5 (80)	1.06	0.666

Abbreviations: Vit-D: vitamin D; CD: Crohn's disease; IFX: infliximab.

**Table 5:** The influence of infliximab trough concentration on clinical outcomes at the week 14 and week 38 treatment in Crohn's disease patients.

Time point	Outcomes	IFX-TC			*Z*	*P*
		Insufficiency	Efficiency	Sufficiency		
Week 14	Biochemical remission	17/24 (70.83)	17/20 (85)	9/9 (100)	-1.949	0.036^∗^
	Clinical remission	18/32 (56.25)	19/24 (79.17)	8/10 (80)	-1.914	0.032^∗^

Week 38	Biochemical remission	11/16 (68.75)	15/19 (78.95)	6/8 (75)	-0.496	0.342
	Clinical remission	17/20 (85)	17/19 (89.47)	7/7 (100)	-0.999	0.245
	Endoscopic remission	16/22 (72.73)	13/18 (72.22)	9/9 (100)	-1.258	0.121
	Endoscopic response	12/22 (54.55)	13/18 (72.22)	9/9 (100)	-2.406	0.009^∗^

Abbreviations: TC: trough concentration; IFX: infliximab; CD: Crohn's disease. ^∗^*P* < 0.05.

## Data Availability

If the data needs to be provided, please contact co-author Huihui Zhang (email: bqedkj@163.com).

## Supplementary Materials

Supplementary 1 Table S1: the influence of infliximab anti-TNF*α* antibody on clinical outcomes at the week 14 and week 38 treatment in Crohn's disease patients.

Supplementary 2 Table S2: demographic characteristics in different vitamin D levels.
