# Supplementary material for: Correlation between Treatment Outcomes and Serum Vitamin D Levels As Well As Infliximab Trough Concentration among Chinese Patients with Crohn's Disease
Source: Gastroenterol Res Pract. 2023 Oct 6;2023:6675401. doi: 10.1155/2023/6675401 (PMC10575748; doi:10.1155/2023/6675401)
Supplement: Supplementary 1 — Table S1: the influence of infliximab anti-TNFα antibody on clinical outcomes at the week 14 and week 38 treatment in Crohn's disease patients. [file 6675401.f1.doc]

Supplement data-table

Table S1: The influence of infliximab anti-TNFα antibody on clinical outcomes at the week 14 and week 38 treatment in Crohn's disease patients

| Time  point | Outcomes | Week 14-ATI | | Z | P |
| --- | --- | --- | --- | --- | --- |
|  |  | + | - |  |  |
| Week  14 | Biochemical remission | 3/3(100) | 40/50(80) | -0.852 | 0.527 |
|  | Clinical remission | 2/4(50) | 43/62(69.35) | -0.799 | 0.38 |
| Week  38 | Biochemical remission | 0/1(0) | 32/42(76.19) | -1.706 | 0.256 |
|  | Clinical remission | 2/2(100) | 39/44(88.64) | -0.499 | 0.792 |
|  | Endoscopic remission | 2/2(100) | 36/47(76.60) | -0.769 | 0.598 |
|  | Endoscopic response | 2/2(100) | 32/47(68.09) | -0.949 | 0.477 |

TC, trough concentration; IFX, infliximab; ATI, anti-TNFα antibody;CD, Crohn's disease;*, P＜0.05
