# Supplementary material for: Correlation between Treatment Outcomes and Serum Vitamin D Levels As Well As Infliximab Trough Concentration among Chinese Patients with Crohn's Disease
Source: Gastroenterol Res Pract. 2023 Oct 6;2023:6675401. doi: 10.1155/2023/6675401 (PMC10575748; doi:10.1155/2023/6675401)
Supplement: Supplementary 2 — Table S2: demographic characteristics in different vitamin D levels. [file 6675401.f2.doc]

| **Table S2: Demographic characteristics in different vitamin D level** | | | | |
| --- | --- | --- | --- | --- |
| **Variables** | **Deficiency** | **Insufficiency** | **Sufficiency** | **P** |
| Number of patients | 54 | 23 | 7 |  |
| Age(y) | 25(21.75~31) | 26(23~34) | 34(27~42) | 0.063 |
| Gender |  |  |  |  |
| Female(%) | 17(31.48) | 4(17.39) | 3(42.86) | 0.321 |
| Height(m)(mean±SD) | 1.68±0.09 | 1.69±0.08 | 1.67±0.08 | 0.833 |
| Weight(Kg)(mean±SD) | 52.35±8.94 | 53.7±8.96 | 54.03±8.18 | 0.779 |
| BMI |  |  |  |  |
| Underweight(%) | 30(55.56) | 11(47.83) | 3(42.86) | 0.499 |
| Normal weight(%) | 24(44.44) | 11(47.83) | 4(57.14) |  |
| Pre-obese(%) | 0(0) | 1(4.35) | 0(0) |  |
| Provinces |  |  |  |  |
| Chongqing(%) | 46(85.19) | 22(95.65) | 5(71.43) | 0.23 |
| Sichuan(%) | 7(12.96) | 1(4.35) | 1(14.29) |  |
| Others(%) | 1(1.85) | 0(0) | 1(14.29) |  |
| Employment situation |  |  |  |  |
| Employed(%) | 29(53.7) | 12(52.17) | 5(71.43) | 0.657 |
| Occupation |  |  |  |  |
| Indoor(%) | 52(96.3) | 21(91.3) | 6(85.71) | 0.329 |
| Marital status |  |  |  |  |
| Unmarried(%) | 36(66.67) | 11(47.83) | 2(28.57) | 0.038 |
| Married(%) | 18(33.33) | 11(47.83) | 4(57.14) |  |
| Divorced(%) | 0(0) | 1(4.35) | 1(14.29) |  |
| Smoking status |  |  |  |  |
| Yes(%) | 13(24.07) | 5(21.74) | 2(28.57) | 1 |
| Education background |  |  |  |  |
| Primary school, n(%) | 0(0) | 0(0) | 1(14.29) | 0.437 |
| Middle school(%) | 7(12.96) | 2(8.7) | 0(0) |  |
| Senior high school(%) | 15(27.78) | 7(30.43) | 2(28.57) |  |
| University and above(%) | 32(59.26) | 14(60.87) | 4(57.14) |  |
| Course of disease mean(IQR) | 2.73(1.65~5.66) | 3.94(1.58~5.44) | 5.69(2~10.38) | 0.373 |
| Clinical symptoms |  |  |  |  |
| Abdominal | 34(62.96) | 10(43.48) | 7(100) | 0.018 |
| Diarrhea | 24(44.44) | 10(43.48) | 1(14.29) | 0.366 |
| Hematochezia | 5(9.26) | 1(4.35) | 0(0) | 0.803 |
| Crissum diseases | 2(3.7) | 2(8.7) | 0(0) | 0.704 |
| Extraintestinal manifestations | 2(3.7) | 4(17.39) | 0(0) | 0.121 |
| Fever | 1(1.85) | 1(4.35) | 0(0) | 0.59 |
| Intestinal surgery，n(%) |  |  |  |  |
| Yes | 44(81.48) | 17(73.91) | 2(28.57) | 0.019 |
| Perianal surgery，n(%) |  |  |  |  |
| Yes | 30(55.56) | 16(69.57) | 1(14.29) | 0.041 |
| IMM |  |  |  |  |
| Yes | 20(37.04) | 7(30.43) | 4(57.14) | 0.498 |
| IMM type |  |  |  |  |
| azathioprine | 17(89.47) | 7(100) | 3(75) | 0.565 |
| methotrexate | 1(5.26) | 0(0) | 1(25) |  |
| thalidomide | 1(5.26) | 0(0) | 0(0) |  |
| Diagnosis age, n (%) |  |  |  |  |
| A1≤16 y | 3(5.56) | 1(4.35) | 0(0) | 0.284 |
| A2 17~40y | 45(83.33) | 19(82.61) | 4(57.14) |  |
| A3 ＞40y | 6(11.11) | 3(13.04) | 3(42.86) |  |
| Disease extent, n (%) |  |  |  |  |
| L1 | 12(22.22) | 5(21.74) | 3(42.86) | 0.402 |
| L2 | 5(9.26) | 3(13.04) | 2(28.57) |  |
| L3 | 32(59.26) | 13(56.52) | 2(28.57) |  |
| L3+L4 | 5(9.26) | 1(4.35) | 0(0) |  |
| L2+L4 | 0(0) | 1(4.35) | 0(0) |  |
| Behavior, n (%) |  |  |  |  |
| B1 | 31(57.41) | 11(47.83) | 3(42.86) | 0.697 |
| B2 | 15(27.78) | 9(39.13) | 2(28.57) |  |
| B3 | 8(14.81) | 3(13.04) | 2(28.57) |  |
| Perianal lesions |  |  |  |  |
| Yes | 35(64.81) | 15(65.22) | 5(71.43) | 1 |
